# Colorimetric Chemosensor for Cu^2+^ and Fe^3+^ Based on a *meso*-Triphenylamine-BODIPY Derivative

**DOI:** 10.3390/s23156995

**Published:** 2023-08-07

**Authors:** Sónia C. S. Pinto, Raquel C. R. Gonçalves, Susana P. G. Costa, M. Manuela M. Raposo

**Affiliations:** Centre of Chemistry, University of Minho, Campus de Gualtar, 4710-057 Braga, Portugal; soniapinto4197@gmail.com (S.C.S.P.); raquelrainha10@hotmail.com (R.C.R.G.);

**Keywords:** BODIPY derivative, colorimetric chemosensor, Fe^3+^, Cu^2+^

## Abstract

Optical chemosensors are a practical tool for the detection and quantification of important analytes in biological and environmental fields, such as Cu^2+^ and Fe^3+^. To the best of our knowledge, a BODIPY derivative capable of detecting Cu^2+^ and Fe^3+^ simultaneously through a colorimetric response has not yet been described in the literature. In this work, a *meso*-triphenylamine-BODIPY derivative is reported for the highly selective detection of Cu^2+^ and Fe^3+^. In the preliminary chemosensing study, this compound showed a significant color change from yellow to blue–green in the presence of Cu^2+^ and Fe^3+^. With only one equivalent of cation, a change in the absorption band of the compound and the appearance of a new band around 700 nm were observed. Furthermore, only 10 equivalents of Cu^2+^/Fe^3+^ were needed to reach the absorption plateau in the UV-visible titrations. Compound **1** showed excellent sensitivity toward Cu^2+^ and Fe^3+^ detection, with LODs of 0.63 µM and 1.06 µM, respectively. The binding constant calculation indicated a strong complexation between compound **1** and Cu^2+^/Fe^3+^ ions. The ^1^H and ^19^F NMR titrations showed that an increasing concentration of cations induced a broadening and shifting of the aromatic region peaks, as well as the disappearance of the original fluorine peaks of the BODIPY core, which suggests that the ligand–metal (1:2) interaction may occur through the triphenylamino group and the BODIPY core.

## 1. Introduction

Metal ions play a crucial role in several biological processes, nutrient cycling, and the functioning of the ecosystem. However, the deregulation of ionic concentrations represents a potential risk to living organisms and the ecosystem, leading to health problems and environmental pollution [1,2,3]. Concerning biological systems, metal ions are considered important elements that regulate and participate in several extra- and intra-cellular processes, such as osmotic pressure regulation, cell signaling (e.g., neurotransmission and muscle contraction), protein and enzyme activity required for oxygen transport, energy production, the regulation of gene expression, and the synthesis of essential molecules [4].

Among several other ions, Cu^2+^ and Fe^3+^ play an important role in various biochemical processes; however, the deregulation of the homeostasis of these cations has been identified as the primary cause of many diseases. For example, high levels of Cu^2+^ can lead to a higher production of reactive oxygen species (ROS) which contributes to the development of different pathologies, such as cancer and neurodegenerative diseases, including Menkes, Wilson, and Alzheimer’s. Moreover, studies have shown that copper levels in the blood serum of cancer patients are considerably above normal values. In contrast, iron deficiency is also associated with several diseases, such as anemia, diabetes, Parkinson’s, and dysfunction of the heart, pancreas, and liver [5,6,7,8]. Apart from that, these elements are non-biodegradable, which is an increased risk for the environment and human health. In this sense, the detection of these two important cations has been a major goal [9,10,11].

Colorimetric sensors are a practical tool for the detection and quantification of these analytes with biological and environmental importance. Compared to other analytical techniques, optical chemosensing displays advantages, including simplicity, speed of response, easy and direct visualization of the results, non-expensive equipment, and high selectivity and sensitivity. For those reasons, investment has been made in the design and synthesis of colorimetric sensors for the detection of these cations of interest. Several chemosensors based on rhodamine, aldazine, carbazole, tetrathiafulvalene spirooxazine, triarylimidazopyridine, and Schiff base core have been developed for the detection of Cu^2+^ and Fe^3+^ [9,10,12,13,14,15,16,17,18,19].

4,4-Difluoro-4-bora-3a,4a-diaza-*s*-indacene, also known as BODIPY, has been recognized as a remarkable chromophore scaffold due to its excellent photophysical properties, such as high molar absorptivity coefficients, narrow and intense absorption bands in the visible range, and great photostability. Furthermore, the core of BODIPY can be functionalized in order to improve its photophysical properties for its intended applications [20,21]. Although several chemosensors based on a BODIPY core have been reported for the detection of Cu^2+^ and Fe^3+^, these sensors detect these cations separately [22,23,24]. Most of these chemosensors also display a fluorimetric response which requires specialized equipment for the visualization of the results. In contrast, chemosensors that are based on a colorimetric response provide direct and real-time detection and identification of the target analyte. To the best of our knowledge, a BODIPY derivative capable of detecting Cu^2+^ and Fe^3+^ simultaneously through a colorimetric response has not yet been described in the literature. Additionally, given the paramagnetic behavior of Cu^2+^ and Fe^3+^ and, consequently, their fluorescence quenching effect, optical sensors with an intense colorimetric response for these analytes are extremely attractive [25,26].

**Figure 1:**
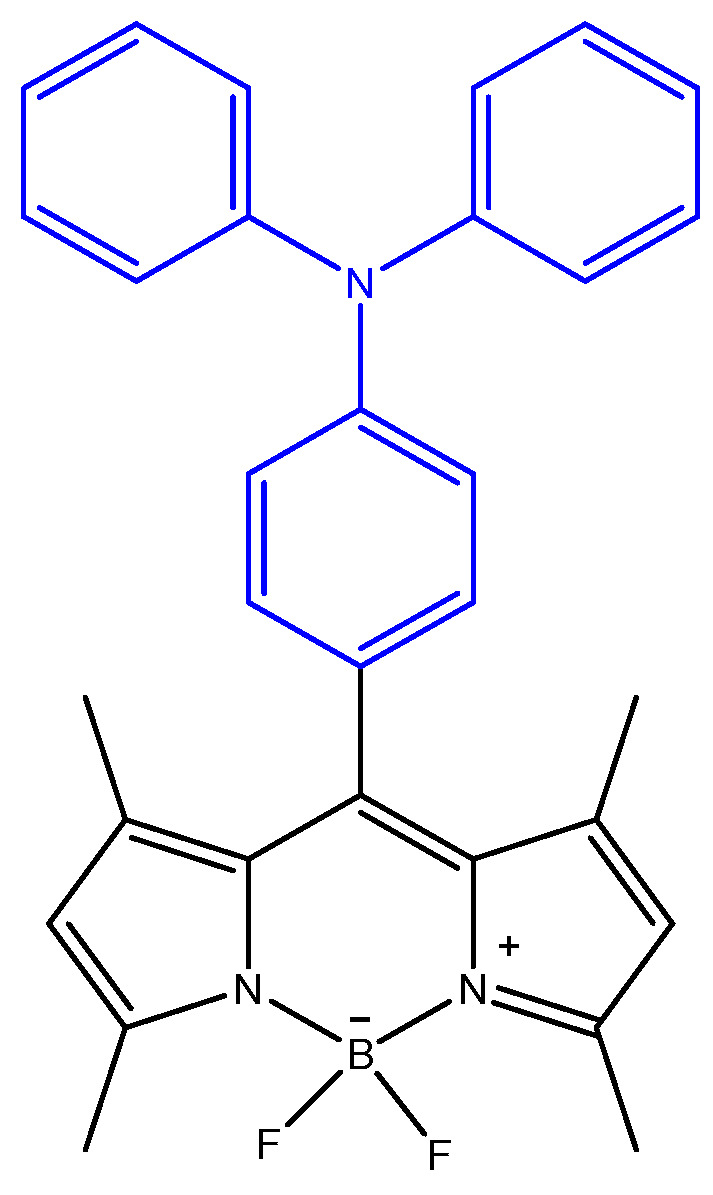
Structure of BODIPY **1**.

In continuation of the research developed by our group [27,28,29,30], we present an evaluation of the ion chemosensory ability of a *meso*-triphenylamine-BODIPY **1** in this work (TPA-BODIPY, Figure 1), which was recently synthesized by us [31,32]. The chemosensory ability was investigated by a preliminary study against several cations and anions with biomedical relevance, followed by spectrophotometric titrations, the Job’s plot method, and ^1^H NMR titrations.

## 2. Materials and Methods

### 2.1. Materials

All absorption measurements were obtained using a Shimadzu UV/3101PC spectrophotometer (200–800 nm). ^1^H NMR analysis was carried out on a Bruker Avance III apparatus at an operating frequency of 400 MHz at 25 °C using the solvent peak as an internal reference. The deuterated solvent used was DMSO-*d*_6_ with 99.8% deuteration degree which contained <0.02% *v*/*v* of water (Euriso-top). All commercial reagents and solvents were used as received. BODIPY **1** was previously synthesized by our research group [31,32].

### 2.2. Synthesis of BODPY Derivative ***1***

2,4-Dimethylpyrrole (1.0 mmol) and 4-(diphenylamino)-benzaldehyde (1.0 mmol) were dissolved in dry dichloromethane (DCM) (100 mL) in the presence of a catalytical amount of trifluoroacetic acid (TFA). The reaction mixture was stirred at room temperature for 50 min. 2,3-Dichloro-5,6-dicyano-1,4-benzoquinone (1.9 mmol) dissolved in dry DCM (100 mL) was added to the reaction mixture, and the stirring time was extended for another 50 min. Triethylamine (16 mmol) was added, followed by treatment with BF_3_.OEt_2_ (26.8 mmol). After stirring for 30 min, the mixture was evaporated under reduced pressure, and the crude residue was purified by dry flash chromatography using a petroleum ether/ethyl acetate (4:1) mixture as the eluent. The pure BODIPY derivative **1** (Figure 1) was obtained as a dark red and orange solid with a 16% yield. ^1^H NMR (400 MHz, CDCl_3_): *δ* = 1.60 (s, 6H, CH_3_-1 and CH_3_-7), 2.57 (s, 6H, CH_3_-3 and CH_3_-5), 6.02 (s, 2H, H-2 and H-6), 7.06–7.19 (m, 9H, 9 × Ar-H), and 7.22–7.27 (m, 5H, 5 × Ar-H) ppm. ^13^C RMN (100.6 MHz, CDCl_3_): *δ* = 14.56, 121.16, 123.30, 123.46, 124.72, 128.881, and 129.45 ppm. MS (ESI) m/z (%): 493 ([M + 2]^+•^, 10), 492 ([M + 1]^+•^, 46), 491 ([M]^+•^, 100), 419 (13), and 274 (20). HRMS (ESI) m/z: [M]^+•^ calculated for C_31_H_28_BF_2_N_3_, 491.2339 was found to be 491.2345.

### 2.3. Photophysical Characterization of BODIPY Derivative ***1***

The photophysical characterization of BODIPY derivative **1** in acetonitrile (1 × 10^−5^ M) was carried out using a standard quartz cells 1 cm optical path. A 1 × 10^−5^ M Rhodamine 6G (*Φ_F_* = 0.95) solution in ethanol was used as a fluorescence standard [33]. Fluorescence was measured after excitation of the compound and as the standard at the maximum absorption wavelength of BODIPY **1**. The relative quantum fluorescence yield of BODIPY **1** was calculated using Equation (1).

(1) $$\Phi_{F_{comp}}=\frac{A_{s}\times F_{comp}\times n_{s}^{2}}{A_{comp}\times F_{s}\times n_{comp}^{2}}\times \Phi_{F_{s}}$$

where *A_s_* and *A_comp_*, *F_s_* and *F_comp_*, and *n_s_* and *n_comp_* correspond to the absorbances, areas under the fluorescence curve, and refractive index value of the solvent of the standard and compound **1**, respectively.

### 2.4. Preliminary Chemosensory Tests

The preliminary study of the chemosensory capacity of the BODIPY **1** (1 × 10^−5^ M) was carried out in acetonitrile (ACN). The cation solutions (Ag^+^, K^+^, Li^+^, Na^+^, Cu^+^, TBT^+^, Cs^2+^, Hg^2+^, Ca^2+^, Co^2+^, Pb^2+^, Mn^2+^, Fe^2+^, Zn^2+^, Ni^2+^, Cd^2+^, Cu^2+^, Pd^2+^, Sn^2+^, Fe^3+^, and Al^3+^) in the form of perchlorate salts, except for Pd^2+^ and Li^+^ whose counter-ion was tetrafluoroborate, were prepared in ACN with a concentration of 1 × 10^−2^ M. These ions were selected due to their biological and environmental interest. Then, 50 equivalents (equiv.) of each ion were added to the solution of the compound, and the color variation of the solutions was visualized under natural light and the fluorescence variation under UV radiation at λ_max_ = 312 nm. The absorption spectrum of BODIPY **1** in the presence of all cations was obtained using 3 mL of BODIPY **1** solution in ACN (1 × 10^−5^ M) and 10 equivalents (30 µL) of each cation in acetonitrile (1 × 10^−2^ M).

### 2.5. Spectrophotometric Titrations, Limit of Detection, and Binding Constant

Spectrophotometric titrations were carried out for Cu^2+^ and Fe^3+^ since these ions induced a relevant optical response. The titrations were performed at room temperature using the solution of **1** in ACN (1 × 10^−5^ M) and the solutions of Cu^2+^ and Fe^3+^ in acetonitrile (1 × 10^−2^ M). A successive addition of each cation was made to 3 mL of compound **1** solution in a standard quartz cuvette. The absorption graphs were collected until the absorbance reached a plateau.

The limit of detection (LOD) for Cu^2+^ and Fe^3+^ was calculated using the slope of the linear zone of absorbance *versus* the ion concentration graph and the standard deviation of 5 replicates of absorbance measurements of the analyte-free solution. According to these data, the LOD was calculated by the following equation (Equation (2)) [25]:

(2) $$LOD=\frac{3\sigma }{k}$$

where *σ* is the standard deviation of the absorbance of the chemosensor without analyte and *k* is the slope of the linear fitting of the calibration curve.

The binding constant of both complexes was determined using UV-vis titration data and the Benesi–Hildebrand equation (Equation (3)) [34,35]:

(3) $$\frac{1}{A-A_{0}}=\frac{1}{K_{a}\left(A_{max}-A_{0}\right){\left[Ion\right]}^{n}}+\frac{1}{A_{max}-A_{0}}$$

where *A*_0_ is the absorbance of **1** in the absence of ion, A is the absorbance of **1** with different concentrations of ion, *A_max_* is the absorbance of **1** at the complete interaction with ion, *n* is the binding stoichiometry of metal, and *K_a_* is the binding constant value. This *K_a_* value was calculated using the ratio of the intercept to the slope of the straight line from the Benesi–Hildebrand plot.

### 2.6. Job’s Plot

The binding stoichiometry of the complexes **1**-Cu^2+^ and **1**-Fe^3+^ was determined using the Job’s plot method. The solutions of both ions and compound **1** were prepared in ACN with a final concentration of 5 × 10^−5^ M. Then, 0.3–2.7 mL of the compound **1** solution was transferred to vials, and the Cu^2+^ and Fe^3+^ solutions were added to each solution of **1** prepared previously to complete a final volume of 3 mL per vial. The UV-vis spectrum of the different samples was recorded at 697/700 nm, and the absorbance values against the molar fractions of each cation—Cu^2+^ and Fe^3+^—were plotted.

### 2.7. ^1^H and ^19^F NMR Titration

NMR titrations were accomplished using a similar sequential addition of ion equivalents to the compound solution. For this process, an NMR glass tube with **1** (2.53 mg) dissolved in 600 μL of DMSO-*d*_6_ or ACN-*d*_3_ and a Cu^2+^ solution (3 × 10^−1^ M) in 1000 μL of DMSO-*d*_6_ or ACN-*d*_3_ were prepared. Then, 0–5 equivalents of Cu^2+^ were sequentially added to the NMR glass tube, and the ^1^H NMR spectrum was recorded after each addition.

## 3. Results and Discussion

### 3.1. Synthesis of BODIPY ***1***

**Scheme 1:**
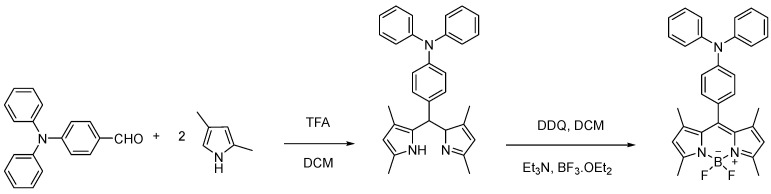
Synthesis of BODIPY derivative **1**.

BODIPY derivative **1** functionalized at the *meso* position with the triphenylamino (TPA) group (Figure 1) was previously synthesized by our research group through two reactional steps at room temperature. The first step involved a condensation reaction of 2,4-dimethylpyrrole and 4-(diphenylamino)-benzaldehyde in dichloromethane (DCM) in the presence of a catalytic amount of trifluoroacetic acid (TFA) to form the dipyrromethane core. The second reactional step consisted of the oxidation of dipyrromethane to dipyrromethene through the addition of a solution of 2,3-dichloro-5,6-diciano-p-benzoquinone (DDQ) followed by a complexation reaction with BF_3_.OEt_2_ in the presence of triethylamine (Scheme 1). Finally, the crude residue was purified using petroleum ether/ethyl acetate (4:1) as eluent through a dry flash chromatography column, and the pure compound was obtained as an orange solid with a 16% yield. Through the ^1^H NMR spectrum, it was possible to confirm the presence of the triphenylamine group at the *meso* position of the BODIPY core, with the characteristic proton signals appearing in the aromatic zone of the spectrum. Additionally, the signals in the ^13^C NMR spectrum and the obtained data from mass spectrometry were in agreement with the expected structure [31,32].

The D-π-A BODIPY molecule comprises a signaling subunit based on the BODIPY core, as an electron acceptor chromophore that is functionalized at position eight with a recognition subunit based on a TPA group which functions as the electron-donating moiety. Additionally, TPA has been previously reported as the binding site of Fe^3+^ through the N atom [36].

### 3.2. Photophysical Characterization of BODIPY Derivative ***1***

The spectroscopic characterization of BODIPY **1** was carried out in acetonitrile solution (1 × 10^−5^ M). The derivative **1** exhibited an intense absorption band (log*ε* = 3.96) at 497 nm, and upon excitation, it showed an emission band at 519 nm. The relative fluorescence quantum yield, which was determined by using Rhodamine 6G in ethanol as a standard (*Φ_F_* = 0.95), was found to be quite low (*Φ_F_* = 0.005), probably due to the rotational freedom and the higher probability of the occurrence of non-radiative relaxation. The Stokes’ shift of BODIPY derivative **1** was relatively short (22 nm, 454,545.45 cm^−1^), which is usual for this class of compounds [37].

### 3.3. Preliminary Chemosensing Study

This preliminary chemosensory study is a simple and quick approach to evaluate the possible interaction between BODIPY **1** and different ions. In this sense, the colorimetric and fluorimetric behavior of BODIPY **1** was studied in acetonitrile (ACN) by the addition of 50 equivalents of biologically and environmentally important cations (Ag^+^, K^+^, Li^+^, Na^+^, Cu^+^, TBT^+^, Cs^2+^, Hg^2+^, Ca^2+^, Co^2+^, Pb^2+^, Mn^2+^, Fe^2+^, Zn^2+^, Ni^2+^, Cd^2+^, Cu^2+^, Pd^2+^, Sn^2+^, Fe^3+^, and Al^3+^) to the compound’s solution. Acetonitrile was used as solvent for this study since it is an aprotic solvent which cannot establish hydrogen bonds and interfere with the recognition system.

**Figure 2:**
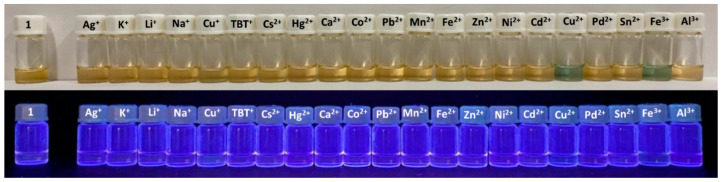
Preliminary chemosensory study of BODIPY **1** in ACN upon interaction with different cations under natural light (**above**), and UV radiation at λ_max_ = 312 nm (**below**).

As shown in Figure 2, the compound **1** solution displays a light yellow color in the absence of any cation. However, upon the addition of 50 equivalents of each ion to the compound solution, a clear color change of the solution from light yellow to blue–green in the presence of Cu^2+^ and Fe^3+^ was observed, while other cations were unable to induce a perceptible color change. In contrast, compound **1** did not exhibit fluorescence emission variation in the presence of any cation. Additionally, the preliminary chemosensory study of compound **1** in the presence of different anions (H_2_PO_4_^−^, CH_3_COO^−^, NO_3_^−^, ClO_4_^−^, HSO_4_^−^, BzO^−^, Br^−^, CN^−^, I^−^, F^−^) was also tested, but no optical response was detected.

**Figure 3:**
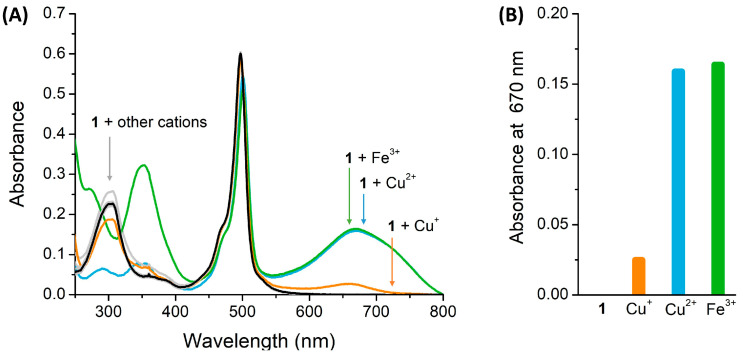
(**A**) Absorption spectra of compound **1** with 10 equivalents of several ions in ACN (**B**) Absorbance data at 670 nm of the interest ions in a bar diagram.

In addition, the absorption spectrum of the compound was plotted in the absence and presence of 10 equivalents of each cation. As depicted in Figure 3A, compound **1** in the absence of ions (in black) shows a maximum absorption band at 497 nm, which corresponds to the light yellow of the compound solution in ACN, and another band at 305 nm, corresponding to the absorption of the TPA moiety [38,39]. The characteristic absorption spectrum of compound **1** suffered significant changes in the presence of 10 equivalents of Cu^2+^ and Fe^3+^, with the appearance of a new band around 670 nm. In contrast, the other cations induced either no or a slight change in the compound’s absorption spectrum. In the case of Cu^+^, the presence of the cation induced the appearance of a new absorption band around 670 nm; however, it does not show a significant absorbance intensity change when compared to Cu^2+^ and Fe^3+^ (Figure 3B), which is in agreement with the results from the preliminary chemosensory ability (Figure 2) where no significant change in the color of the solution of compound **1** was observed in the presence of 50 equivalents of Cu^+^. Moreover, the absorption band that appears around 355 nm in the presence of Fe^3+^ (in green) corresponds to the self-absorption of Fe^3+^ [40]. Based on these results, compound **1** shows an interesting potential as a selective colorimetric chemosensor of Cu^2+^ and Fe^3+^.

### 3.4. Spectrophotometric Titrations

According to the results obtained in the preliminary evaluation of the chemosensory capacity of BODIPY **1**, spectrophotometric titrations in acetonitrile were carried out to further investigate the efficiency of BODIPY **1** toward Cu^2+^ and Fe^3+^ detection. Thus, increasing amounts of each cation (0–10 equivalents) were added to the compound’s solution; simultaneously, the corresponding absorption spectrum was obtained.

**Figure 4:**
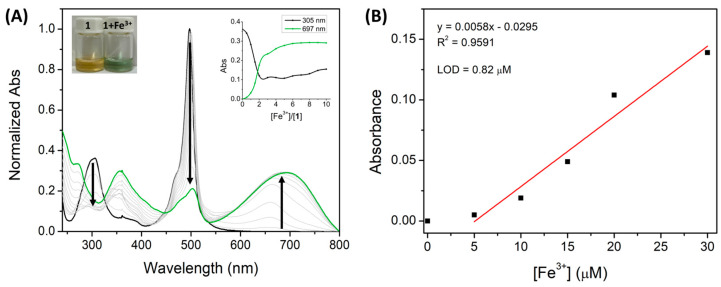
(**A**) Normalized UV-vis absorption titration of **1** (1 × 10^−5^ M) with Fe^3+^ (0–100 μM) in ACN. (The insert represents the normalized absorption at 492 nm as a function of Fe^3+^ equivalents). (**B**) The linear relationship between the Fe^3+^ concentration and the absorbance of **1** (at 697 nm).

**Figure 5:**
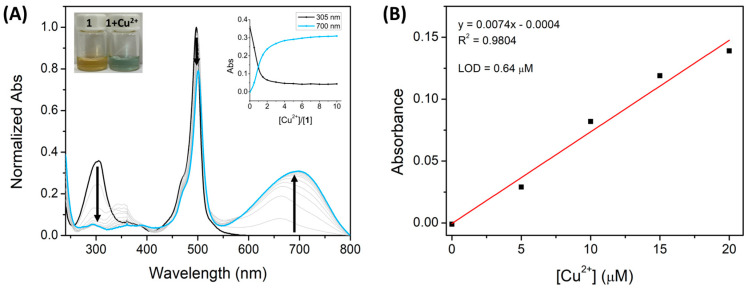
(**A**) Normalized UV-vis absorption titration of **1** (1 × 10^−5^ M) with Cu^2+^ (0–100 μM) in ACN. (The insert represents the normalized absorption at 492 nm as a function of Cu^2+^ equivalents). (**B**) The linear relationship between the Cu^2+^ concentration and the absorbance of **1** (at 700 nm).

The titration graphs allowed us to follow the changes in the compound’s absorption bands while the number of equivalents of each cation was increasing. After the addition of increasing amounts of Cu^2+^ and Fe^3+^, a progressive decrease in the absorption band of 305 nm was observed, while a new red-shifted band appeared at 697 nm and 700 nm for Cu^2+^ and Fe^3+^, respectively. This reflects a color change in the compound’s solution from light yellow to blue–green (Figure 4A and Figure 5A). Moreover, the appearance of a new absorption band around 700 nm in the presence of these cations can be attributed to the complex formation between **1** and Cu^2+^/Fe^3+^ ion. These results indicate that Cu^2+^ and Fe^3+^ may have identical coordination behavior with compound **1**. Concerning the peak at 492 nm, the decrease was significantly more prominent in the titration with Fe^3+^ when compared to Cu^2+^. The complexation between cation-ligand **1** reached the maximum variation of the absorption band intensity with only 10 equivalents of each cation which proved the high sensitivity of BODIPY **1** toward Cu^2+^ and Fe^3+^.

Additionally, the corresponding absorbance calibration curve was obtained as a function of the Cu^2+^ and Fe^3+^ concentration (Figure 4B and Figure 5B). Both calibration curves of Cu^2+^ and Fe^3+^ exhibited good linear relationships, with correlation coefficients of R^2^ = 0.9804 and 0.9591, respectively.

Therefore, Cu^2+^ and Fe^3+^ can be quantified within this range. According to these data, the limit of detection (LOD) was calculated using the slope of the linear zone of absorbance versus the ion concentration graph.

Moreover, the maximum acceptable copper and iron level (20.5 μM and 5.4 μM, respectively) in drinking water, as stated by the World Health Organization (WHO) and the U.S. Environmental Protection Agency (EPA), and the normal range of copper and iron in whole blood is between 13–21 µM and 7–29 µM, respectively [41,42,43,44]. These reference values are higher than the LOD values of **1** for Cu^2+^ (0.63 µM) and Fe^3+^ (0.82 µM) and are within the linear range of the calibration curve. The BODIPY derivative **1**, like other chemosensors, has a low limit of detection for Cu^2+^ (LOD = 0.63 µM) and Fe^3+^ (LOD = 0.82 µM). However, many reported colorimetric sensors in the literature display a detection signal for these cations at a lower absorption wavelength (ranging from 292 to 570 nm) [12,19,45,46,47,48,49,50], whereas our compound has a detection signal around 700 nm. This feature is an advantage for the potential quantitative monitoring of Cu^2+^ and Fe^3+^ ions in environmental/biological samples since water and some biomolecules have a strong self-absorption ranging from 250 to 580 nm [51,52]. Thus, these interferences may be avoided at longer wavelengths, resulting in a higher sensitivity detection of the target cations. These results suggest a good potential of compound **1** as a colorimetric chemosensor for these cations in environmental and biological samples.

### 3.5. Binding Stoichiometry and Constant

**Figure 6:**
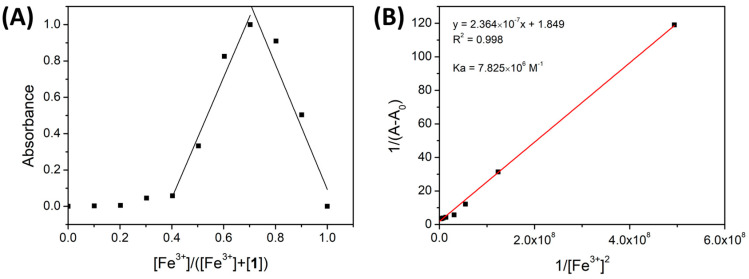
(**A**) A Job’s plot of **1**-Fe^3+^ in ACN. The absorbance was recorded at 693 nm. (**B**) A Benesi-Hildebrand diagram based on a spectrophotometric titration of **1** with Fe^3+^ at 697 nm.

**Figure 7:**
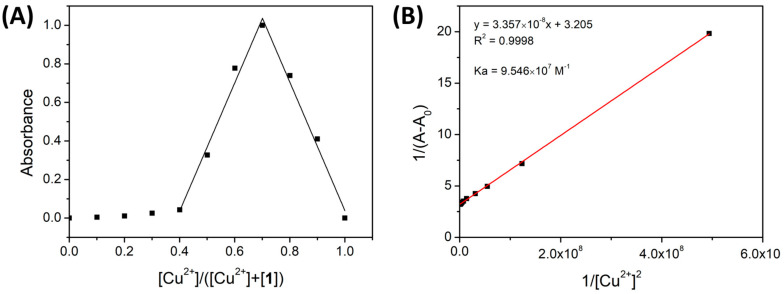
(**A**) Job’s plot of **1**-Cu^2+^ in ACN. The absorbance was recorded at 700 nm. (**B**) Benesi–Hildebrand diagram from spectrophotometric titration of **1** with Cu^2+^ at 700 nm.

The binding stoichiometry of the complexes **1**-Cu^2+^ and **1**-Fe^3+^ was determined using a Job’s plot [53] with absorption bands at 697 nm and 700 nm, respectively. The corresponding absorbance was plotted as a function of the mole fraction of the cation (Figure 6A and Figure 7A). In both cases, the highest absorbance was reached at cation 0.7 mole fraction, indicating the formation of a 1:2 complex between ligand **1** and metal.

The binding constant values were determined by the Benesi–Hildebrand method using the UV-vis titration data. The absorbance variations in the presence of Cu^2+^ and Fe^3+^ (697 nm and 700 nm, respectively) (1/(*A* − *A*_0_)) were plotted as a function of 1/[ion]^2^, and the binding constant (*K_a_*) was calculated using the ratio of the intercept to the slope of the graph.

As shown in Figure 6B and Figure 7B, both Benesi–Hildebrand plots exhibited good linear relationships with correlation coefficients of R^2^ = 0.9998 and 0.998. This good fitting confirms the 1:2 complex formation and corroborates the Job’s plot results. The Ka of **1**-Cu^2+^ and **1**-Fe^3+^ was calculated to be 7.825 × 10^6^ M^−1^ and 9.546 × 10^7^ M^−1^, respectively, indicating a strong complexation between compound **1** and Fe^3+^/ Cu^2+^ ions and a higher binding affinity of Fe^3+^ toward **1** than Cu^2+^.

### 3.6. ^1^H and ^19^F NMR Titrations

**Figure 8:**
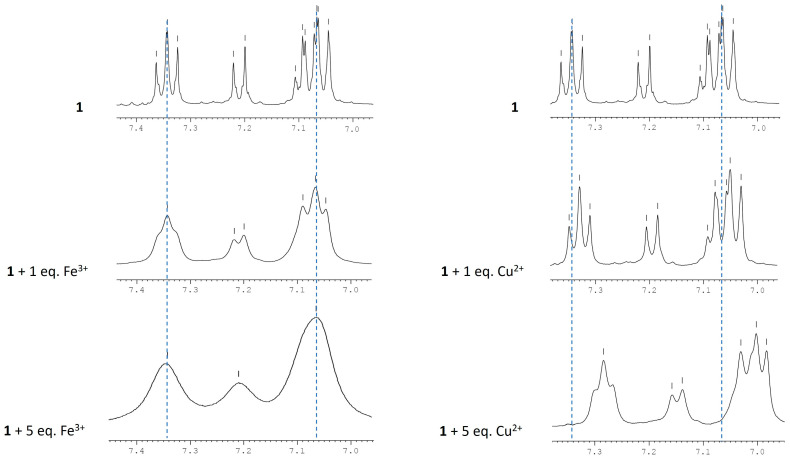
^1^H NMR spectra of **1** in the presence of increased amounts of Fe^3+^ and Cu^2+^ in DMSO-*d*_6_.

To corroborate the previous findings and investigate the binding site of Cu^2+^ and Fe^3+^, the ^1^H and ^19^F NMR titrations of **1** with Cu^2+^ and Fe^3+^ were carried out. In this study, the concentration of compound **1** was fixed, while the concentration of the cation was gradually increased (0–5 eq.), and the ^1^H and ^19^F NMR spectra were recorded. As depicted in Figure 8, the proton peaks in the aromatic region ascribed to the TPA moiety became broader, and the proton signal at 7.36–7.04 ppm shifts to 7.28–6.98 ppm upon the sequential addition of Cu^2+^. Considering that the paramagnetic behavior of Cu^2+^ and Fe^3+^ affects the relaxation times of protons, and consequently, the frequency of protons near the ions’ binding region, these results point to an interaction with the TPA moiety, which can possibly occur through the coordination of the cations with the N atom of the triphenylamino group [36].

To avoid the presence of the broad water peak in the protons’ region of the BODIPY core, we also performed the ^1^H NMR titrations in deuterated acetonitrile (Figure S1). It was observed a breadth, decrease of intensity, and shift of the aromatic protons in the presence of 1 equivalent of Cu^2+.^ Moreover, the proton peaks of the BODIPY core were also affected by the presence of the cation. As shown in Figure S1, the singlets of H-2 and H-6 at 6.11 ppm and CH_3_-3 and CH_3_-5 at 2.48 ppm split in two different peaks, respectively. In contrast, the singlet of CH_3_-1 and CH_3_-7 at 1.59 ppm only suffered a slight shift. These results may indicate the loss of the BODIPY core’s symmetry.

**Figure 9:**
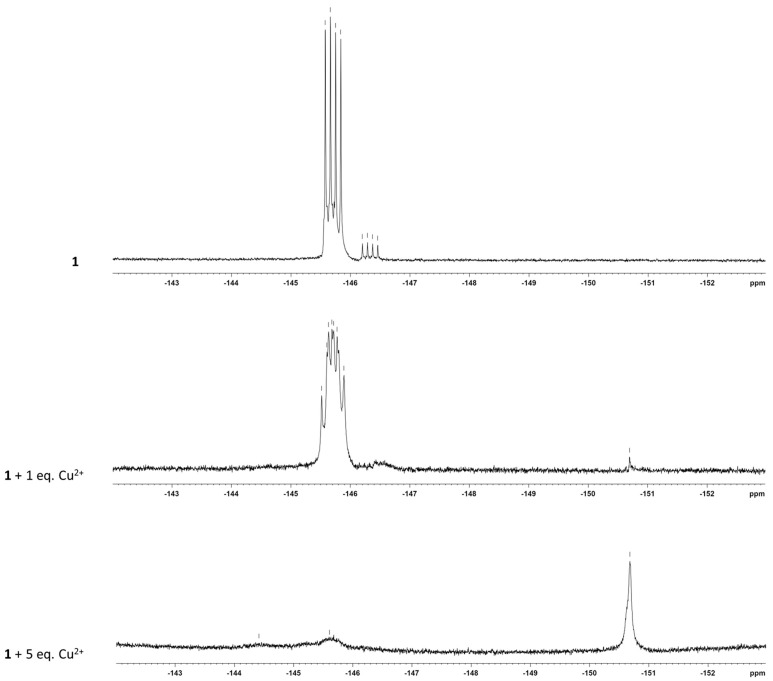
^19^F NMR spectra of BODIPY **1** in the absence and presence of increase amount of Cu^2+^ in ACN-*d*_3_.

Additionally, the ^19^F NMR spectrum was recorded to gain more insight into a possible interaction between the cations and the BODIPY core. As shown in Figure 9, it was observed a doublet of quartets for each fluorine atom due to the ^19^F-^19^F coupling and ^19^F-^11^B coupling [54] in the absence of the cation, whereas in the presence of one equivalent of Cu^2+^, a broadening of the peak at −145.69 ppm and a complete disappearance of the peak at −146.33 ppm was observed. The addition of five equivalents of the cation induced the appearance of a new peak at −150.68 ppm together with the loss of the peak at −145.69 ppm. This suggests the formation of a new species of boron and fluoride, not complexed by the N atom.

Furthermore, we performed the ^19^F NMR of BF_3_OEt_2_ to compare the fluorine chemical shifts of a BF species not complexed by N. As shown in Figure S2, two peaks appear at −150.44 ppm and −151.95 ppm which are similar to the new peak that appears at −150.68 ppm after the addition of five equivalents of the cation. These data suggest that the interaction of Cu^2+^ with the BODIPY core may change the environment around the F and could be explained by the formation of a BF species dissociated from one of the N or even from both N.

All these findings suggest the coordination of the cations with BODIPY **1** may be occurring through the nitrogen atom of the TPA moiety and simultaneously with the fluorine atoms of the BODIPY core [55], which is consistent with the formation of a 1:2 complex between ligand **1** and the metal proved by the Job’s plot experiment.

## 4. Conclusions

In this work, a *meso*-triphenylamine-BODIPY derivative was reported for the highly selective detection of Cu^2+^ and Fe^3+^. In the preliminary chemosensing study, this compound showed a significant color change from yellow to blue–green in the presence of Cu^2+^ and Fe^3+^. With only one equivalent of cation, a change in the absorption band of the compound and the appearance of a new band around 700 nm were observed. Furthermore, only 10 equivalents of Cu^2+^/Fe^3+^ were needed to reach the absorbance plateau in the UV-visible absorption titrations. Compound **1** showed an excellent sensitivity toward Cu^2+^ and Fe^3+^ detection with LODs of 0.63 µM and 1.06 µM, respectively. The binding constant calculation indicated a strong complexation between compound **1** and Cu^2+^/Fe^3+^ ions. The ^1^H and ^19^F NMR titrations suggested that the ligand-metal interaction may occur through the nitrogen atom of the TPA moiety and simultaneously with the fluorine atoms of the BODIPY core. Hence, BODIPY derivative **1** has demonstrated great potential as a chemosensor for the rapid, selective, and sensitive detection of Cu^2+^ and Fe^3+^.

## Data Availability

Not applicable.

## Supplementary Materials

The following supporting information can be downloaded at: https://www.mdpi.com/article/10.3390/s23156995/s1, Figure S1: ^1^H NMR spectra of BODIPY **1** in the absence and presence of one equivalent of Cu^2+^ in ACN-*d*_3_; Figure S2: ^19^F NMR spectra of BF_3_OEt_2_ in ACN-*d*_3_.
