# Supplementary material for: Colorimetric Chemosensor for Cu2+ and Fe3+ Based on a meso-Triphenylamine-BODIPY Derivative
Source: Sensors (Basel). 2023 Aug 7;23(15):6995. doi: 10.3390/s23156995 (PMC10422517; doi:10.3390/s23156995)
Supplement: Supplementary file 1 [file sensors-23-06995-s001.zip › sensors-2523456-supplementary.pdf]

Supporting Information

Colorimetric Chemosensor for Cu^{2+} and Fe^{3+} Based on a *meso*-Triphenylamine-BODIPY Derivative

Sónia C. S. Pinto, Raquel C. R. Gonçalves, Susana P. G. Costa and M. Manuela M. Raposo *

Centre of Chemistry, University of Minho, Campus de Gualtar, 4710-057 Braga, Portugal
*Correspondence: mfox@quimica.uminho.pt

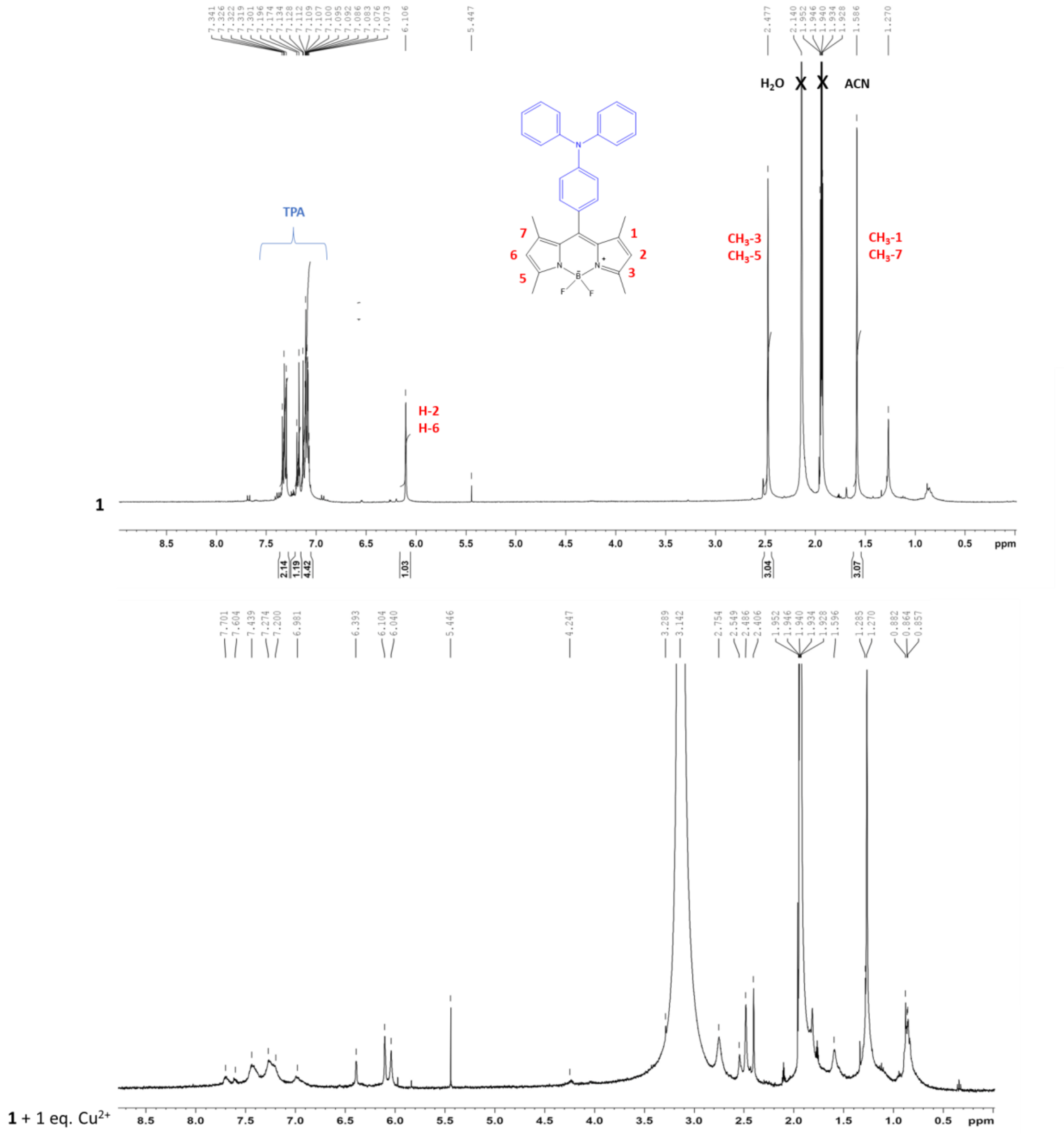

Figure S1. ^{1}H NMR spectra of BODIPY **1** in the absence and presence of one equivalent of Cu^{2+} in ACN-*d*3.

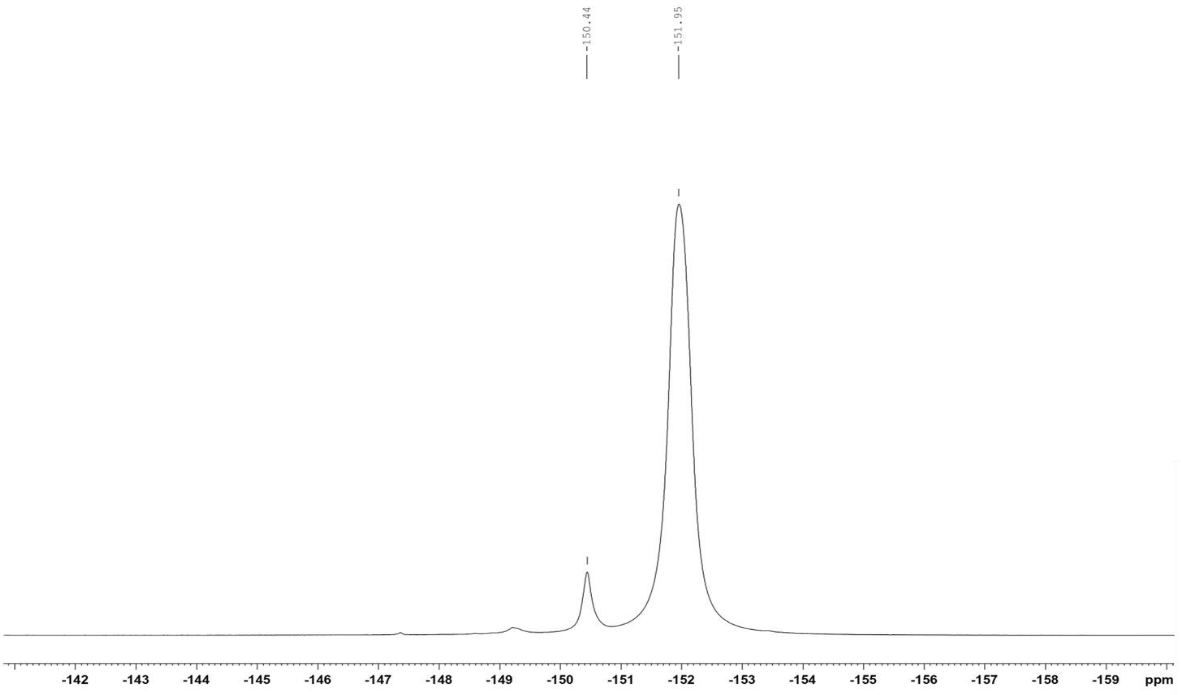

Figure S2. ^{19}F NMR spectra of BF_3OEt_2 in ACN-d_3.
